# Nylon 6 electrospun nanofibers mat as effective sorbent for the removal of estrogens: kinetic and thermodynamic studies

**DOI:** 10.1186/1556-276X-9-353

**Published:** 2014-07-15

**Authors:** Fei-Fei Qi, Yang Cao, Min Wang, Fei Rong, Qian Xu

**Affiliations:** 1 Key Laboratory of Environmental Medicine Engineering, Ministry of Education, School of Public Health, Southeast University, 210009 Nanjing, China; 2 Suzhou Key Laboratory of Environment and Biosafety, 215123 Suzhou, China; 3 Zibo Municipal Center for Disease Control and Prevention, 255026 Zibo, China

**Keywords:** Nylon 6 electrospun nanofibers mat, Adsorption, Estrogens, Kinetics, Thermodynamics

## Abstract

Nylon 6 electrospun nanofibers mat was prepared via electrospinning for the removal of three estrogens, namely, diethylstilbestrol (DES), dienestrol (DS), and hexestrol (HEX) from aqueous solution. Static adsorption as well as the dynamic adsorption was evaluated by means of batch and dynamic disk flow mode, respectively. The kinetic study indicated that the adsorption of the target compounds could be well fitted by the pseudo-second-order equation, suggesting the intra-particle/membrane diffusion process as the rate-limiting step of the adsorption process. The adsorption equilibrium data were all fitted well to the Freundlich isotherm models, with a maximum adsorption capacity values in the range of 97.71 to 208.95 mg/g, which can be compared to or moderately higher than other sorbents published in the literatures. The dynamic disk mode studies indicated that the mean removal yields of three model estrogens were over 95% with a notable smaller amount of adsorbent (4 mg). Thermodynamic study revealed that the adsorption process was exothermic and spontaneous in nature. Desorption results showed that the adsorption capacity can remain up to 80% after seven times usage. It was suggested that Nylon 6 electrospun nanofibers mat has great potential as a novel effective sorbent material for estrogens removal.

## Background

Synthetic estrogens are some of the most potent endocrine-disrupting chemicals (EDCs) found in municipal wastewater, despite of low concentration (ng/L) [1]. Given pervasive contamination and the highly toxic nature of synthetic estrogens, there is considerable interest in the development of techniques to remove these compounds from contaminated water. Since these compounds are hydrophobic compounds of low volatility, adsorption plays an important role in their removal [2-4].

In principle, the heart of the sorption technique is the sorbent material. Several kinds of materials have been used as adsorbent for estrogens, such as carbon nanomaterials [5], activated charcoal [6,7], fullerene-containing membranes [8], multi-walled carbon nanotubes [9], granular activated carbon, chitin, chitosan, ion-exchange resin and a carbonaceous adsorbent prepared from industrial waste [10,11], iron (hydr)oxide-modified activated carbon fibers [12], etc. These materials showed good performance for the removal of estrogens from wastewater. However, they are suffering a common problem that it needs a next separation process from the wastewater, which will increase the operation cost. Thus, further research is needed to find new adsorbents with optimized disposal process and high removal performance.

Recently, there is a growing interest on sorbents based on nanofibers for their characteristics [13]. As reported by the literatures, polymer nanofibers obtained by electrospinning show excellent heavy-metal ions and organic pollutants removal ability from water [14-16]. However, to our knowledge, no reports using electrospun nanofibers as adsorbent for the removal of estrogens have appeared up to now.

Nylon 6 is a general chemical material, consisting of amide groups which are separated by methylene sequences, where nonpolar interactions are expected between hydrophobic compounds and the methylene chains of Nylon 6. Our previous research, using the Nylon 6 electrospun nanofibers mat as solid-phase extraction (SPE) sorbent, has demonstrated the highly effective extraction nature of the Nylon 6 nanofibers mat for nonpolar and medium polarity EDCs, such as natural and synthetic estrogens [17,18], bisphenol A [19], and phthalate esters [20,21] in environmental water. It is indicated from the results of our work that the extremely large surface-to-volume ratio and numerous micropores make nanofibers mat a promising high-performance adsorbent material that can achieve a larger specific surface and more active sites for adsorption, compared with microscale adsorbents. Accordingly, the adsorption of the target compounds is facilitated and a small amount nanofiber (2 ~ 3 mg) is sufficient [17-21]. Furthermore, some researchers have indicated that polymer fiber mat as the adsorbent could avoid the subsequent separation process [22]. All the facts mentioned above revealed that the Nylon 6 electrospun nanofibers mat has a great potential as an efficient adsorbent.

The objective of this research is to go deeper on the basis of our previous work and investigate the detailed adsorption characteristics and thermodynamics of estrogen removal by Nylon 6 electrospun nanofibers. Diethylstilbestrol (DES), dienestrol (DS), and hexestrol (HEX) were chosen as the model target estrogens. The static adsorption as well as the dynamic adsorption was evaluated by means of batch and dynamic disk flow mode. Kinetic and thermodynamic studies of removal of estrogens were investigated based on the experimental data for the understanding of the adsorption characteristic. Results from this study were used to evaluate the feasibility of Nylon 6 electrospun nanofibers as sorbent for estrogen removal in real-wastewater treatment.

## Methods

### Chemicals

High-purity standards of three estrogens including DES, DE, and HEX were purchased from Sigma Company, St. Louis, MO, USA. Methanol, acetonitrile, and acetone of HPLC grade used for analysis were obtained from Tedia Inc, Fairfield, OH, USA. Cresol, formic acid, hydrochloric acid, and sodium hydroxide were analytical reagent grade, which were purchased from Chemical Reagent Factory, Shanghai, China. Nylon 6 material was purchased from DebioChem, Nanjing, China.

### Preparation of Nylon 6 nanofibers mat

The Nylon 6 nanofibers mat was fabricated by electrospinning described previously [17-21]. The procedure was briefly as follows. An appropriate amount of Nylon6 was dissolved in a composite solvent of formic acid and *m*-cresol (6:4, *v*/*v*). This solution was loaded into a glass syringe (volume 5 mL). The glass syringe was fitted to a stainless needle (diameter 0.5 mm) with a flat tip connected to the anode. With an interval of 20 cm, a grounded aluminum foil was served as the collection screen, and a voltage of 15 kV (DW-P403-1 AC high-voltage generator, Dongwen Factory, Tianjing, China) was applied between the tip and the aluminum foil. The rate of movement of the syringe was controlled and fixed at 0.5 mL/h by a syringe pump (model TCI-I, SLGO, Beijing, China). A dense mat of Nylon 6 nanofibers with its thickness in the range of 70 to 200 μm was collected on the aluminum foil while the electronspun time was 2 to 8 h. A scanning electron microscope (SEM, Hitachi S-3000 N, Tokyo, Japan) was utilized to characterize the Nylon 6 nanofibers mat. The surface-to-volume ratio of Nylon 6 nanofibers was measured by the ASAP 2020 Accelerated Surface Area and Porosimetry system (Micromeritics Instrument Corporation, Norcross, USA).

### Instrument and analytical conditions

The quantitative method of the three estrogens was established in our previous work [18]. Briefly, a Thermo Finnigan TSQ Quantum Ultra tandem mass spectrometer equipped with an electrospray ionization (ESI) source (San Jose, CA, USA), a Finnigan surveyor LC pump, and an auto sampler were used for LC-MS/MS analysis. Data acquisition was performed with Xcalibur 1.1 software (Thermo-Finnigan, San Jose, CA, USA). Peak integration and calibration were carried out using LC Quan software (Thermo-Finnigan).

An ODS column (250 mm × 4.6 mm i.d., particle size 5 mm) was used for analysis at 35°C. A mixture of methanol and water (80:20, *v*/*v*) at a flow rate of 1.0 mL/min was used as the mobile phase, and the split ratio was 4:1. The ionization of each compound was tested in negative multiple reaction monitoring (MRM) mode. Nitrogen was used as the sheath gas (35 psi) and the auxiliary gas (5 psi). The capillary temperature was 350°C, and the spray voltage was 3.5 kV. The injection volume was 10 μL throughout the study.

### Adsorption experiments

Adsorption of three model estrogens from aqueous solutions was established by batch adsorption experiments. Nylon 6 nanofibers mat (1.5 mg) was immersed into 50 mL estrogen solution of a desired concentration in 100-mL glass conical flasks with cover, the solution was standing for 6 h to establish adsorption equilibrium kinetic experiments (0 to 6 h) and adsorption isotherm (initial concentration 0.1 to 2.0 mg/L), and thermodynamic studies (273 to 323 K) on adsorption were studied. Based on the results of our previous work, 10.0 mg/L estrogen solution was chosen for the determination of maximum adsorption capacity at 298 K. The temperature effect on the kinetics of estrogen adsorption was also investigated. All the adsorption isotherm experiments were carried out at temperature of 298 K. Fifty-microliter samples were withdrawn from the solutions in the course of adsorption and were collected at regular intervals of time (0, 1, 2, 3, 4, 5, and 6 h) for three model estrogens analysis. HPLC-MS/MS method discripted as above was applied to quantify the adsorbents concentrations. The removal percentage of three model estrogens can be calculated by the following equation:

(1) $$\%\mathrm{removal}=\;C_{o}-C_{e}/C_{o}\;\times \;100$$

The equilibrium adsorption capacity (*q*_e_) was determined using the following equation:

(2) $$q_{e}=V\;\times \;\frac{C_{o}-C_{e}}{m}$$

where *C*_o_ is the initial concentration of estrogens in solution (mg/L) and *C*_e_ is the equilibrium concentration (mg/L). *m* is the mass of adsorbent (g), and *V* is the volume of solution (L).

The adsorption capacity was calculated by the following equation:

(3) $$q_{t}=V\;\times \;\frac{C_{o}-C_{t}}{m}$$

where *q*_*t*_ is the adsorption capacity at time *t*, *C*_o_ is the initial concentration of estrogens in solution (mg/L), *C*_t_ is the concentration at time t (mg/L), *m* is the mass of adsorbent (g), and *V* is the volume of solution (L).

**Figure 1:**
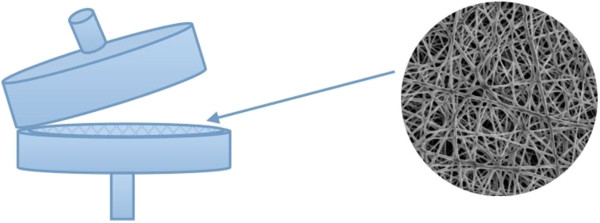
Home-made disk filter device for dynamic disk mode adsorption studies.

Independent blank experiments found that there was no estrogen adsorption from the glass conical flasks and all experiments above were performed in triplicate.The dynamic disk mode adsorption studies were carried out in a home-made disk filter device (Figure 1) at 298 K to aid in ascertaining the practical applicability of the adsorbent in the real system. One piece of Nylon 6 nanofibers mat was accurately cut into a circular shape with a diameter of approximately 20 mm and attached tightly to the filter. The nanofibers mat was preconditioned with 200 μL methanol and 200 μL water once each. One hundred milliliter estrogen solution at a certain initial concentration was pumped upwards through the disk filter device using a peristaltic pump. The effects of various variables in process such as adsorbent amount (1.0 to 5.0 mg, depended on the thickness of the Nylon 6 nanofiber mat) and flow rate (0.5 to 4.0 mL/min) on removal yields were assessed and optimized at the constant initial concentration (5.0 mg/L). The maximum dynamic adsorption capacities of estrogens on Nylon 6 nanofiber mat were evaluated under the optimum dynamic flow conditions via breakthrough initial concentration (1.0 to 20.0 mg/L).

### Desorption experiment

For desorption studies, 1.5 mg Nylon 6 nanofibers mat was first contacted with 50 mL 2 mg/L estrogens for 6 h at 298 K. Then the adsorbent was eluted by 0.5 mL methanol/water (80:20, *v*/*v*, i.e., mobile phase for HPLC separation) for 20 min. Before the second adsorption, Nylon 6 nanofibers mat was washed with 0.5 mL water on a magnetic stirrer at 200 rpm. The above procedure was repeated for seven times to test the reusability of the adsorbent.

## Results and discussion

### Morphology of the nanofibers mat

The morphology of Nylon 6 nanofibers mat was studied by SEM; the results are shown in Figure 1. We can see that the surface of Nylon 6 nanofibers was smooth, the average diameter is about 200 nm, and the average specific surface of Nylon 6 fibers was 23.90 m^2^/g.

### Adsorption kinetics

**Figure 2:**
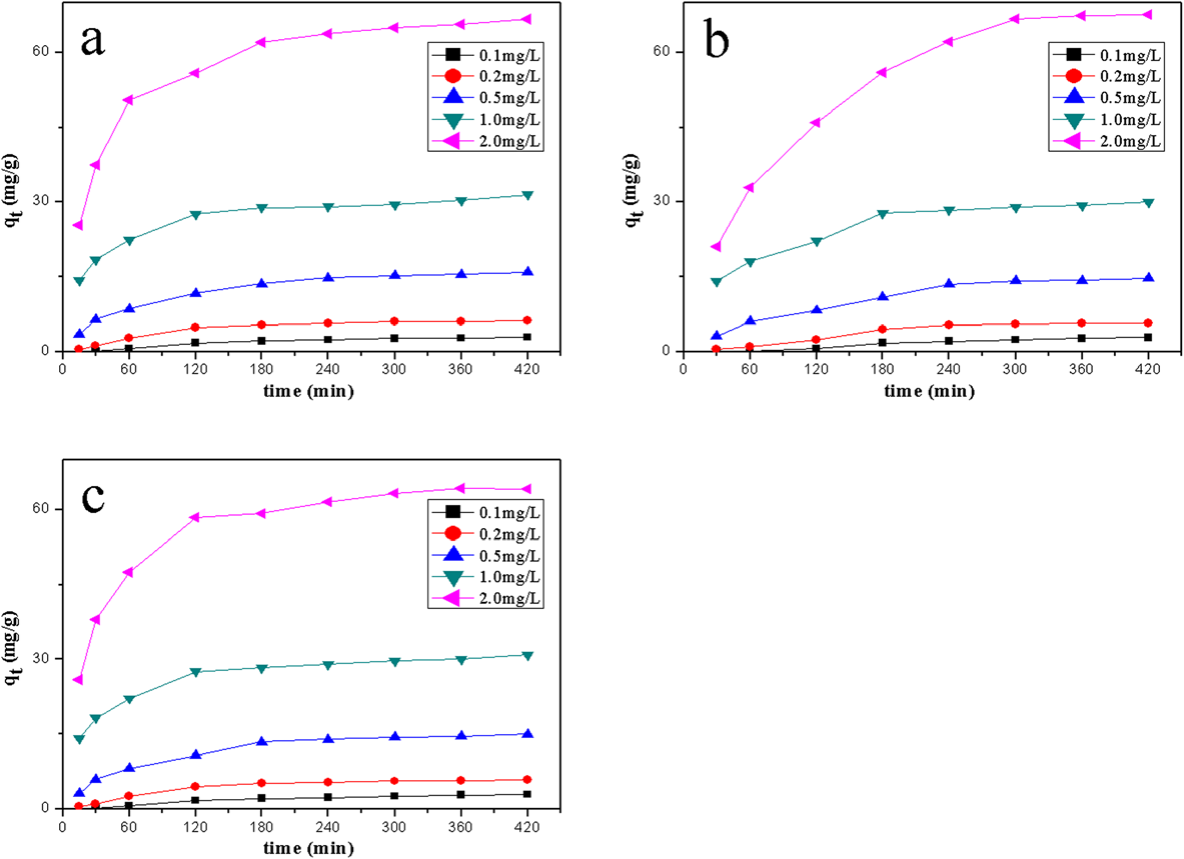
Time and concentration to the adsorption of DES (a), DE (b), and HEX (c).

The effect of adsorption time on the adsorption capacity at different initial solution concentration is shown in Figure 2. The results indicated that the adsorption capacity of the three estrogens increased with an increase in adsorption time until equilibrium was reached between the adsorbents and estrogens solution. The equilibrium time of the three estrogens increased from 120 to 180 min as the initial solution concentration increased from 0.1 to 2.0 mg/L. And the equilibrium capacity DES, DE and HEX increased from 2.98 to 68.88 mg/g, 3.21 to 66.66 mg/g, 3.01 to 64.22 mg/g, respectively, with the initial concentrations of estrogens solution increase from 0.1 to 2.0 mg/L.

In order to better understand the adsorption behaviors, parameters from two commonly used kinetic models, namely, the pseudo-first-order and the pseudo-second-order, were fit to experimental data to examine the adsorption kinetics of three estrogens uptake by Nylon 6 nanofibers mat. These two kinetic models are used to describe the adsorption of solid/liquid systems, which can be expressed in the linear forms as Eqs. (4) and (5), respectively [23]:

(4) $$\mathrm{lg}\left(q_{e}-q_{t}\right)=\mathrm{lg}q_{e}-\frac{K_{1}}{2.303}$$

(5) $$\frac{t}{q_{t}}=\frac{1}{K_{2}q_{e^{z}}}+\frac{1}{q_{e}}t$$

where K_1_ and K_2_ are the pseudo-first-order and second-order rate constants, respectively.

**Figure 3:**
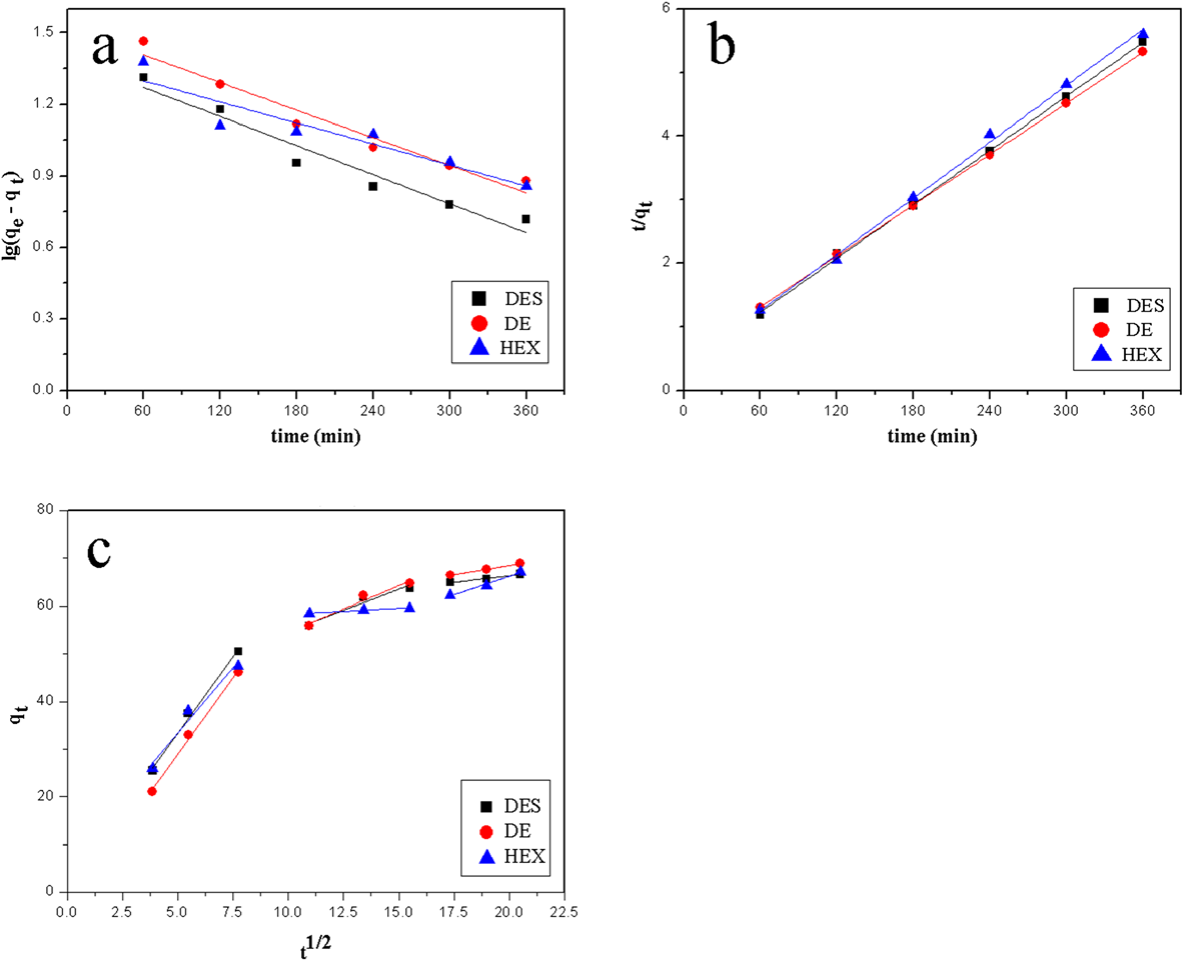
The adsorption kinetic plots for the adsorption of three estrogens.

The adsorption kinetic plots for the adsorption of three estrogens are shown in Figure 3, and the obtained kinetic parameters are summarized in Table 1.

**Table 1:** Adsorption kinetic model rate constants for three estrogens adsorption on Nylon 6 nanofibers mat at different temperatures

**Target compound**	***q***_**e,exp **_**(mg/g)**	**Pseudo-first-order model**			**Pseudo-second-order model**		
		**K**_**1 **_**(min**^**−1**^**)**	***q***_**e,cal **_**(mg/g)**	***R***_**1**_^**2**^	**K**_**2 **_**(g/mg min)**	***q***_**e,cal **_**(mg/g)**	***R***_**2**_^**2**^
DES	68.88	0.00467	24.65	0.937	0.000525	70.9219	0.993
DE	66.66	0.00437	33.27	0.942	0.000345	75.1879	0.999
HEX	64.22	0.00338	24.30	0.844	0.000532	71.4285	0.997

The values of the correlation coefficients (*R*^2^) clearly indicated that the adsorption kinetics closely followed the pseudo-second-order model rather than the pseudo-first-order model (the results draw the same conclusion under initial concentration 0.1 to 2.0 mg/L; to be concise, kinetic parameters obtained from initial concentration 2.0 mg/L are presented in Figure 3 and Table 1 only). The pseudo-second-order rate constant (K_2_) of DES, DE, and HEX decreased from 0.00239 to 0.000525 g/mg/min, 0.00123 to 0.000346 g/mg/min, and 0.00130 to 0.000533 g/mg/min, respectively, with an increase in initial concentration from 0.1 to 2.0 mg/L. Moreover, the *q*_e_, calculated values obtained from the pseudo-second-order kinetic model appeared to be very close to the experimentally observed values than the values from the pseudo-first-order kinetic model. The results accordingly indicated that the adsorption kinetics of three estrogens adsorbed onto the Nylon 6 nanofiber mat closely followed the pseudo-second-order kinetic model (Figure 3a) rather than the pseudo-first-order kinetic model (Figure 3b), suggesting that intra-particle/membrane diffusion process was the rate-controlling step of the adsorption process [23]. So, it was necessary to analyze the intra-particle/membrane diffusion model in order to describe the adsorption process more clearly.

The Weber-Morris intra-particle/membrane diffusion model has often been used to determine if intra-particle/membrane diffusion is the rate-limiting step [24,25]. According to this model, a plot of *q*_*t*_ versus *t*^1/2^ should be linear if intra-particle/membrane diffusion is involved in the adsorption process, and it is essential for the plots to cross the origin if the intra-particle/membrane diffusion is the sole rate-controlling step [23]. In this work, the plot did not pass through the origin; instead, three linear portions were obtained (Figure 3c); and this suggested that adsorption occurred in three phases, involving diffusion to the external surface, intra-particle/membrane diffusion or gradual adsorption being the rate-controlling stage, and the final equilibrium stage where the intra-particle/membrane diffusion slowed down due to the extremely low solute concentration in solution [26]. As the plots did not pass through the origin, intra-particle/membrane diffusion was not the only rate-limiting step. It might be estimated that the surface diffusion assisted by agitation or oscillation could benefit to the adsorption process, considering the condition of static adsorption in this work was just standing to establish adsorption equilibrium.

### Adsorption isotherm

**Figure 4:**
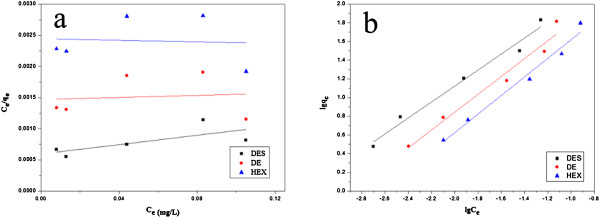
The adsorption isotherms of the three estrogen removal by Nylon 6 nanofibers mat at 298 K.

Adsorption isotherms indicated a distribution of adsorbate between solution and adsorbent when adsorption process reaches an equilibrium state. The adsorption isotherms of the three estrogen removal by Nylon 6 nanofiber mat at 298 K are shown in Figure 4. Two well-known models of Freundlich and Langmuir isotherms were used to fit the equilibrium data, and the correlation coefficient (*R*^2^) obtained was used to evaluate the fitness of the two models.

As the description in the literature [23], the Freundlich isotherm is used to describe the adsorption onto the heterogeneous surface of an adsorbent and is applicable to both monolayer (chemisorption) and multilayer adsorption (physisorption). The linear form of Freundlich equation is expressed as:

(6) $$\mathrm{lg}q_{e}=lgK_{F}+\frac{1}{n}\mathrm{lg}C_{e}$$

where K_F_ and n are Freundlich isotherm constants related to adsorption capacity and adsorption intensity, respectively and *C*e is the equilibrium concentration (mg/L).

The Langmuir isotherm model, on the other hand, describes monolayer adsorption on a uniform surface with a finite number of adsorption sites [23]. No further sorption can take place at the same site once it has been filled before. When all the adsorption sites on the surface are saturated, the maximum adsorption will be achieved. The linear form of the Langmuir isotherm model is defined as:

(7) $$\frac{C_{e}}{q_{e}}=\frac{C_{e}}{q_{\text{max}}}+\frac{1}{K_{L}q_{\text{max}}}$$

Where K_L_ is the Langmuir constant related to the energy of adsorption and *q*_max_ is the maximum adsorption capacity (mg/g).

The values of these parameters are summarized in Table 2. The higher values of correlation coefficient reveal that Freundlich model better fitted the isotherm data compared to the Langmuir model.

**Table 2:** Langmuir and Freundlich constants for the adsorption of three estrogens on Nylon 6 nanofibers mat

**Target compound**	**Langmuir constants**			**Freundlich constants**		
	**K**_**L **_**(h**^**−1**^**)**	**q**_**max n**_**(mg/g)**	**R**_**1**_^**2**^	**K**_**F**_	**n**	**R**_**2**_^**2**^
DES	0.94	162.60	0.204	683.439	1.1695	0.9389
DE	6.01	166.66	0.3707	564.937	1.0484	0.9574
HEX	1.69	227.27	0.1369	409.355	1.0068	0.9743

The maximum adsorption capacity of DES, DE, and HEX obtained from the experiment was 208.95, 135.21, and 97.71 mg/g, respectively. The results of adsorption of EDCs obtained from the literatures based on other kinds of sorbent materials were also selected as references for comparative studies, and the comparative information was presented in Table 3. The maximum adsorption capacity of Nylon 6 nanofibers mat for three estrogens obtained in our study is found to be comparable or moderately higher than that of many other corresponding sorbent materials, although the target EDCs were different, because the relative study of removal of the three model EDCs chosen in this study has not published so far. Moreover, it was noteworthy that a small amount nanofiber (1.5 mg) was sufficient for the highly effective adsorption in our work.

**Table 3:** Comparison with other sorbent materials in literatures

**Sorbent materials**	**Target EDCs**	**Amount of sorbents**	**Maximum adsorption capacity or constant**	**Literatures**
Carbon nanomaterials	17α-Ethinyl estradiol, bisphenol A	-	50 to 600 mg/g	5
Activated charcoal	17α-Ethinyl estradiol	0.25 g	7.47 μg/g	6
Activated charcoal	Estriol	0.25 g	3.34 μg/g,	7
Fullerene-containing membranes	Estrone	-	582 ng	8
Multi-walled carbon nanotubes	Estriol, 17α-ethinyl estradiol	50 mg	0.52 μg/g, 5.59 μg/g	9
Carbonaceous adsorbent	Estrone, 17β-estradiol	1.0 g	9290 mL/g, 12200 mL/g	10
Chitin	Benzo(a)antracene, β-estradiol, bisphenol A	10 mg	42.9 to 84 mg/g	11
Iron(hydr)oxide modified activated carbon fibers	Estrone, 17α-ethinyl estradiol	-	1.8 mg/g	12
Nylon 6 nanofibers mat (this work)	Diethylstilbestrol, dienestrol, and hexestrol	1.5 mg	208.95 mg/g, 135.21 mg/g, 97.71 mg/g	

The possible reason might be the large surface area and high porosity of Nylon 6 nanofibers mat. Furthermore, as the primary chemical structure of nylon consists of amide groups separated by methylene sequences, nonpolar interactions are expected between hydrophobic estrogens and the methylene chains of nylon, and meanwhile, the hydrophilic amide groups are expected to enhance the water molecule movement into the sorbent, improving mass transfer and the chance for uptake. The higher adsorption capacity of the adsorbent used in this study may be coming from these properties of Nylon 6 nanofibers mat.

### Adsorption thermodynamics

The adsorption of the estrogens on the Nylon 6 nanofiber mat was studied at temperature range of 273 to 323 K to determine the thermodynamic parameters, from which the changes in standard enthalpy (∆H^0^, kJ/mol), standard entropy (∆S^0^, kJ/mol K), and standard free energy (∆G^0^, kJ/mol) due to the transfer of unit mole of solute from solution onto the solid-liquid interface can be obtained. The values of ∆H^0^ and ∆S^0^ were calculated using the following equations [27]:

(8) $$\Delta G^{0}=-RT\ln K_{d}$$

(9) $$\Delta G^{0}=\Delta H^{0}-T\Delta S^{0}$$

where R (8.314 J/mol K) is the universal gas constant, *T* (K) is the absolute solution temperature, and *K*_d_ is distribution adsorption coefficient calculated from the following equation [27]:

(10) $$K_{d}=\frac{C_{o}-C_{e}}{C_{e}}\cdot \frac{V}{m}$$

where *C*_o_ is the initial concentration (mg/L), *C*_e_ is the equilibration concentration after adsorption (mg/L), *V* is the volume of the solution (L), and *m* is the dose of the membrane (g). From Eqs. (8) and (9), the van’t Hoff equation was obtained as:

(11) $$\ln K_{d}=\frac{\Delta S^{0}}{R}-\frac{\Delta H^{0}}{\mathrm{RT}}$$

**Figure 5:**
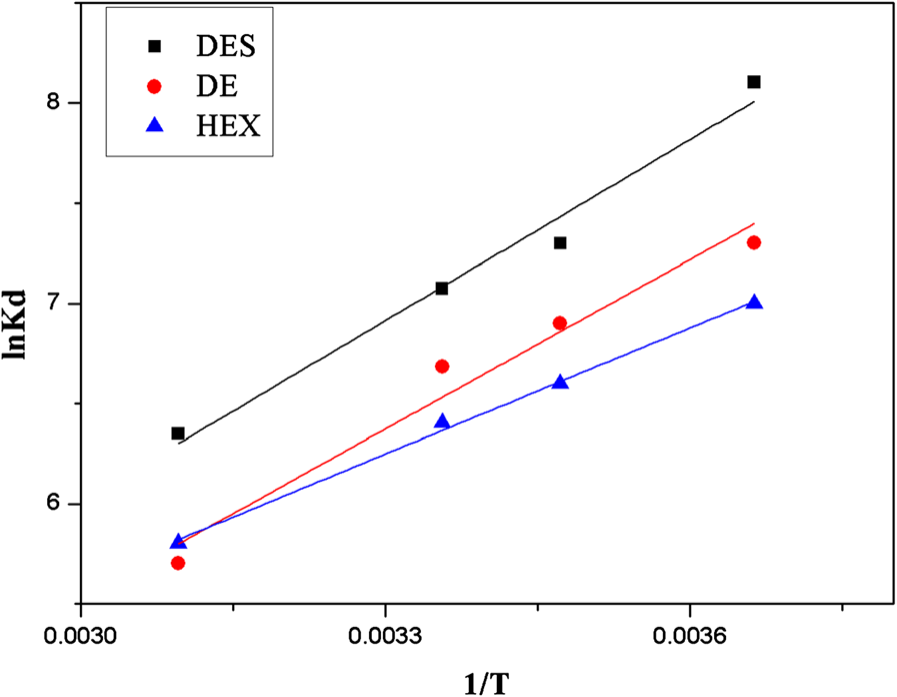
**Plot of lnK**_**d **_**versus 1/T for the estimation of thermodynamic parameters.**

As shown in Figure 5, the plot of lnK_d_ versus 1/T gave a straight line with a slope of ∆H^0^ and an intercept of ∆S^0^. The values of these thermodynamic parameters measured at different temperatures are listed in Table 4.

**Table 4:** Adsorption thermodynamics

**Target compound**	**Temperature (K)**	**∆G**^**0 **^**(kJ/mol)**	**∆H**^**0 **^**(kJ/mol)**	**∆S**^**0 **^**(kJ/mol K)**
DES	273	−18.38	−25.04	−0.025
	288	−17.47		
	298	−17.52		
	323	−17.05		
DE	273	−16.57	−23.42	−0.024
	288	−16.52		
	298	−16.56		
	323	−15.31		
HEX	273	−15.87	−17.43	−0.006
	288	−15.86		
	298	−15.85		
	323	−15.57		

Negative values of ∆G^0^ of the three estrogens indicated spontaneous adsorption and the degree of spontaneity of the reaction decrease with increasing temperature. Because the physical sorption energies are in the range of 0 to −20 kJ/mol and the chemisorption energies in the range of −80 to −400 kJ/mol [28]. The interaction between the three estrogens and Nylon 6 nanofibers mat can be considered as a physical adsorption rather than chemisorption. The negative values of ∆H^0^ indicated that the adsorption process of estrogens on Nylon 6 nanofiber mat was exothermic process. The negative values of ∆S^0^ indicated the decreased randomness at the solid/solution interface during the adsorption of three estrogens in aqueous solution on the nanofibrous membrane.

### Dynamic disk mode studies

Continuous adsorption trials in dynamic flow mode were performed in a home-made disk filter device for the removal of three model estrogens in 100 mL solution. Since the adsorption performance of adsorbents usually depends on available sorbent amount for adsorption, the effect of the Nylon 6 nanofibers mat amount was examined in the range of 1.0 to 5.0 mg (the initial concentration was 5.0 mg/L and flow rate was 1.0 mL/min). The results indicated that the amount of adsorbent strongly influenced estrogens adsorption yield. The removal yields of DES, DS, and HEX increased from 70.15 ± 1.93% to 97.59 ± 2.26%, 62.47 ± 1.96% to 96.72 ± 1.81%, and 60.32 ± 2.23% to 96.26 ± 1.68%, respectively, with an increase in the adsorbent amount from 1.0 to 4.0 mg, and the variations of removal for target contaminants using 5.0 mg nanofibers were not remarkable. The higher adsorption yields for higher adsorbent amount are due to the increase of more available binding sites for the adsorption. And then, after a certain point (4.0 mg), the adsorption yield stayed nearly constant may be due to the saturation of binding sites on the adsorbent surface. Therefore, 4.0 mg of the Nylon 6 nanofibers mat was found to be optimum of the further dynamic flow mode adsorption.

The effect of the flow rate on the estrogen adsorption in continuous mode was also investigated. The flow rate of estrogens solution was varied from 0.5 to 4.0 mL/min while the initial concentration (5.0 mg/L) and adsorbent amount (4.0 mg) were kept constant. It was found that the flow rate strongly influenced estrogen uptake capacity, and lower flow rates favored estrogen adsorption. The maximum removal yields were obtained at flow rates of 0.5 and 1.0 mL/min (*p* > 0.05). The adsorption capacity significantly decreased with increased flow rate from 2.0 to 4.0 mL/min (*p* < 0.05). This was due to a decrease in the residence time of estrogens within the Nylon 6 nanofibers mat at higher flow rates. This caused a weak distribution of the liquid inside the mat, which leaded to a lower diffusivity of the adsorbates to the binding sites for the adsorption. Therefore, removal yields of DES, DS, and HEX decreased from 97.95 ± 1.28% to 75.13 ± 2.14%, 96.55 ± 1.46% to 79.37 ± 1.95%, and 96.85 ± 1.62% to 74.65 ± 2.74%, respectively, with an increase in the flow rate from 1.0 to 4.0 mL/min. The optimal flow rate for estrogens adsorption was chosen as 1.0 mL/min in this study, given an overall consideration of adsorption efficiencies and the cost of the increment of the treatment time.

If the amount of adsorbates was larger than breakthrough adsorption amount of adsorbent materials, target compounds could flow away with solution. In order to obtain high removal efficiency, breakthrough amount should be investigated. Under the optimum conditions, the breakthrough amount was investigated by pumping 100 mL solution with initial concentration of the three target estrogens in the range of 1.0 to 20.0 mg/L through the disk filter device. The results indicated that satisfactory removal yields (above 90%) were obtained during 1.0 to 15.0 mg/L. When the initial concentration was increased to 20.0 mg/L, a drop about 11.29% to 14.76% of removal yields of all the three target estrogens was occurred. The marked decline indicated the adsorption breakthrough of Nylon 6 nanofibers mat. According to the experimental results, the breakthrough initial concentration of all the three estrogens was 15.0 mg/L, while the removal yields of DES, DS, and HEX were 97.55 ± 1.36%, 95.13 ± 1.65%, and 93.37 ± 1.49%, respectively. Therefore, the maximum dynamic adsorption capacity of DES, DS, and HEX by Nylon 6 nanofibers mat was calculated as 365.81, 356.74, and 350.13 mg/g for DES, DS, and HEX, respectively. It was evident that highly dynamic estrogen adsorption performance could be obtained using Nylon 6 nanofibers mat as sorbent material.

### Desorption performance and reusability of Nylon 6 nanofibers mat

**Figure 6:**
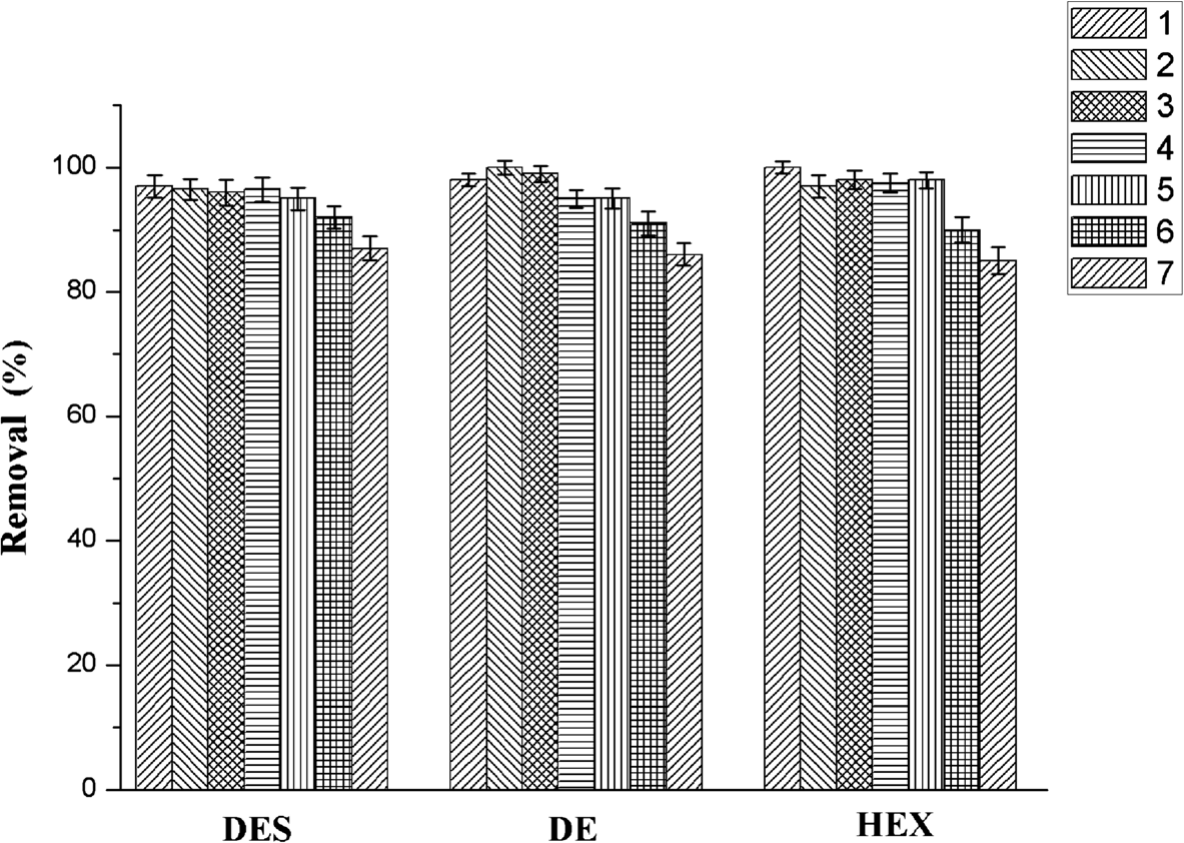
**Reusability of Nylon 6 nanofibers mat (*n*** **= 3).**

As shown in Figure 6, the Nylon 6 nanofibers mat-loaded estrogens were regenerated and present better reuse performance. The estrogen adsorption capacity still remained over 80% after seven times usage. It is clear that the variations of removal yields of target compounds are not obvious for the first six times but were reduced in the seventh time. Therefore, it could be concluded that one mat can be used six times for high-performance adsorption.

## Conclusions

Adsorption technology plays an important role in pollutant removal in environmental water. The key research is to find new adsorbents and clear the detailed adsorption characteristics. This study investigated the kinetics and thermodynamics characteristics of estrogen removal by Nylon 6 electrospun nanofibers for the first time, with an expectation of taking advancement in the feasibility of applications of nanofiber-based adsorption technique for contaminated water treatment. It is suggested by our experimental results that adsorption based on Nylon 6 electrospun nanofibers mat has great potential for estrogen removal in wastewater treatment, moderately higher than other sorbents published in the literatures. Furthermore, a small amount nanofiber is sufficient and regenerated readily and presents better reuse performance.

## Competing interests

The authors declare that they have no competing interests.

## Authors’ contributions

QX designed the experiments. FQ and MW carried out all of the experiments. YC and FR wrote the paper. All authors read and approved the final manuscript.
